# CXCL5 as a potential novel prognostic factor in early stage non-small cell lung cancer: results of a study of expression levels of 23 genes

**DOI:** 10.1007/s13277-014-1605-x

**Published:** 2014-02-06

**Authors:** Oksana Kowalczuk, Tomasz Burzykowski, Wieslawa Ewa Niklinska, Miroslaw Kozlowski, Lech Chyczewski, Jacek Niklinski

**Affiliations:** 1 Department of Clinical Molecular Biology, Medical University of Bialystok, 13 Waszyngtona St, 15-267 Bialystok, Poland; 2 Hasselt University, Diepenbeek, Belgium; 3 Department of Statistics and Medical Informatics, Medical University of Bialystok, Bialystok, Poland; 4 Department of Histology and Embryology, Medical University of Bialystok, Bialystok, Poland; 5 Department of Thoracic Surgery, Medical University of Bialystok, Bialystok, Poland; 6 Department of Medical Pathomorphology, Medical University of Bialystok, Bialystok, Poland

**Keywords:** Non-small cell lung cancer, Prognosis, Molecular markers, CXCL5

## Abstract

As the current staging system is imprecise for estimating prognosis of early stage non-small cell lung cancer (NSCLC), it is important to identify other methods for selecting high-risk patients after failed surgical treatment. The aim of the study was to evaluate the expression of 23 genes as putative prognostic markers in early stage NSCLC. The study was performed on 109 pairs of tumor and matched unaffected lung tissue surgical specimens taken from stage I and II NSCLC patients. We evaluated the mRNA level of 23 genes using the real-time PCR method. The difference in the expression between the tumor and normal tissue for each gene was analyzed using a general linear model. The influence of gene expression on survival was analyzed by using the proportional hazards model. Eighteen out of the 23 genes showed statistically significant differences in expression between the tumor and non-tumor tissue. For 12 genes (*ITGB1*, *ITGB3*, *CXCL1*, *CXCL8*, *CXCL9*, *CXCL10*, *CXCL11*, *CXCR3*, *CXCR4*, *TNF*, *CHKA*, *AGFG1*, and *CTC1*), the expression was lower, and for six genes (*ITGA5*, *IL8*, *IL6*, *CXCL2*, *CXCL3*, and *CXCL12*), it was higher in the tumor tissue as compared to the matched normal tissue. Expression changes were more pronounced in squamous cell carcinomas than in adenocarcinomas or large cell carcinomas. Of all the analyzed genes, only *CXCL5* was found to statistically significantly (*p* = 0.04) influence both overall and disease-free survival. Among the 23 genes previously suggested to be relevant for early staged NSCLC patients’ postoperative outcome, only *CXCL5* showed a statistically significant prognostic effect.

## Introduction

Lung cancer is the most common cause of cancer deaths worldwide, and approximately 80 % of the patients have non-small cell lung cancer (NSCLC). Current mode of lung cancer therapy mainly depends on the TNM disease staging and tumor histological classification, and the treatment-of-choice for early stage NSCLCs constitutes radical surgery.

However, about 30 to 50 % of early stage NSCLC patients (in stages I and II) die within 5 years after radical surgical treatment as a consequence of disease recurrence or metastasis. Results of recent studies have shown some benefit of adjuvant chemotherapy in surgically treated NSCLC patients with 5-year survival advantage of 4–15 % [1]. However, the factors that might help to prospectively identify patients who will most likely benefit from any or specific type of postoperative chemotherapy remain unknown, especially among individuals with stages I–IIA disease.

It has been suggested that evaluation of molecular biomarkers for prediction and prognostics could improve the management of patients with NSCLC [2]. Recent advances in genomics and proteomics of lung cancer have established new candidate markers with potential clinical value. Based on the results of high-throughput microarray and real-time PCR data, several prognostic gene expression signatures have been created to stratify patients for appropriate treatment; however, the proposed gene sets differ significantly from one another. Thus, further studies on gene expression signature as well as verification of previously reported data have to be performed [3].

The quantitative real-time PCR (qRT-PCR) seems to be the most appropriate method for both the microarray-based prognostic gene expression signatures validation and their further practical application in the clinics [4]. Recently, the capabilities of qRT-PCR have been expanded with the development of low density gene expression arrays that allow simultaneous examination of multiple, user-defined genes [5].

In the current study, we evaluated the expression of 23 genes reported previously as putative prognostic markers in NSCLC. The genes used for evaluation were defined according to a number of microarray-based studies (three genes: AGFG1/HRB, STC1, SLC2A1 that overlap between NSCLC prognostic signatures), comprehensive real-time PCR analyses, and/or protein-evaluating assays [6–17]. The selected genes are listed in Table 1. We used low density microarray approach to simultaneously evaluate the expression of all the analyzed genes and to compare gene expression level in tumor and corresponding non-tumor lung tissues. We also analyzed the association between the expression level of the particular genes and the overall survival (OS) and disease-free survival (DFS).

**Table 1:** List of the genes analyzed in the study

No.	Gene symbol	Gene Id^a^	Gene product official name (aliases)	Gene product functions	References
1.	*AGFG1*/*HRB*	5175	Arf-GAP domain and FG repeats containing protein 1	Probably mediates nucleocytoplasmic transport	[6, 7]
2.	*STC1*	11373	Stanniocalcin 1	Probably regulates calcium/phosphate transport and metabolism	[6, 8]
3.	*SLC2A1*	11005	Solute carrier family member 2 (facilitated glucose transporter), member 1	A major glucose transporter in the mammalian blood–brain barrier	[6, 8, 9]
4.	*CHKA*	1937	Choline kinase alpha	Catalyzes the first step in phosphatidylcholine biosynthesis and contributes to phosphatidylethanolamine biosynthesis	[10]
5.	*CXCL1*	4602	Chemokine (C-X-C motif) ligand1 (melanoma growth stimulating activity, alpha)	Recruits neutrophil polymorphonuclear leukocytes at sites of inflammation; stimulates angiogenesis	[11, 12]
6.	*CXCL2*	4603	Chemokine (C-X-C motif) ligand 2 (melanoma growth stimulating activity, beta)	Recruits neutrophil polymorphonuclear leukocytes at sites of inflammation; stimulates angiogenesis	[11]
7.	*CXCL3*	4604	Chemokine (C-X-C motif) ligand 3 (melanoma growth stimulating activity, gamma)	Recruits neutrophil polymorphonuclear leukocytes at sites of inflammation; stimulates angiogenesis	[11, 12]
8.	*CXCL5*	10642	Chemokine (C-X-C motif) ligand5(epithelial-derived neutrophil activating protein 78 (ENA-78))	Involved in neutrophil activation; stimulates angiogenesis	[11]
9.	*CXCL6*	10643	Chemokine (C-X-C motif) ligand 6 (granulocyte hemotactic peptide-2 (GCP-2))	Is a chemoattractant for neutrophil granulocytes; stimulates tumor cell growth	[11]
10.	*CXCR2*	6027	Chemokine (C-X-C motif) receptor 2 (interleukin-8 receptor, beta)	Membrane receptor that binds chemokines CXCL6 and CXCL8 with high affinity	[11]
11.	*CXCR3*	4540	Chemokine (C-X-C motif) receptor 3	Membrane receptor that binds chemokines CXCL9, CXCL10, and CXCL11	[11]
12.	*CXCR4*	2561	Chemokine (C-X-C motif) receptor 4	Membrane receptor specific for chemokine CXCL12/SDF-1. Important in the metastatic behavior of solid tumors	[11, 13, 14]
13.	*CXCL9*	7098	Chemokine (C-X-C motif) ligand 9 (monokine induced by gamma interferon (MIG))	Is a Th1-cell attracting chemokine; blocks angiogenesis	[11, 12]
14.	*CXCL10*	10637	Chemokine (C-X-C motif) ligand 10 (10 kDa interferon-gamma-induced protein (IP-10))	Is chemotactic for monocytes and T\ lymphocytes, has pleiotropic effects on these cells, and blocks angiogenesis	[11]
15.	*CXCL11*	10638	Chemokine (C-X-C motif) ligand 11 (0Interferon-inducible T cell alpha chemoattractant (I-TAC))	Is a chemoattractant to interleukin-activated T cells; blocks angiogenesis	[11]
16.	*CXCL12*	10672	Chemokine (C-X-C motif) ligand 12 (Stromal cell-derived factor 1 (SDF-1))	Is a chemoattractant to T lymphocytes and monocytes	[11, 14]
17.	*IL10*	5962	Interleukin-10 (cytokine synthesis inhibitory factor (CSIF))	Inhibits the synthesis of a number of cytokines including interferon-gamma, IL-2, IL-3,TNF, and Gm-CSF produced by activated macrophages and by helper T cells	[15, 16]
18.	*IL6*	6018	Interleukin-6 (interferon, beta 2(IFNB2))	A cytokine that functions in inflammation and the maturation of B cells and is a potent inducer of the acute phase response	[15]
19.	*IL8*	6025	Interleukin-8 (Chemokine (C-X-C motif) ligand 8(CXCL8))	One of the major mediators of the inflammatory response; stimulates angiogenesis	[12, 15]
20.	*TNF*	11892	Tumor necrosis factor (TNF superfamily, member 2)	A multifunctional pro-inflammatory cytokine involved in the regulation of a wide spectrum of cellular processes, including proliferation, differentiation, and apoptosis	[15]
21.	*ITGA5*	6141	Integrin, alpha 5 (Fibronectin receptor, alpha polypeptide)	A member of integrin family of membrane receptors involved in cell adhesion	[17]
22.	*ITGB3*	6156	Integrin, beta 3 (platelet glycoprotein IIIa, AntigenCD61)	A member of integrin family of membrane receptors involved in cell adhesion	[17]
23.	*ITGB1*	6153	Integrin, beta 1 (Fibronectin receptor)	A member of integrin family of membrane receptors involved in cell adhesion	[17]

^a^According to HUGO Gene Nomenclature Committee (HGNC)

## Materials and Methods

### Patients and samples

The study was performed on 109 pairs of tumor and matched unaffected lung tissue specimens obtained from early stage NSCLC (stages I and II) patients who underwent a curative surgery at the Bialystok Medical University Hospital. All patients gave the written informed consent for specimen collection and clinicopathological data processing. The study was approved by the ethics committee of the university.

All patients underwent complete resection of the tumor and mediastinal lymph nodes and were followed up for at least 3 years or until death. None of them received chemo- or radiotherapy before or after the surgery. The OS was estimated as the time from the date of the surgery to the date of death (event) or of the last control visit (censoring). The DFS was defined as the time from the date of surgery to the date of disease relapse or death (events), whichever occurred first, or to the date of the last visit (censoring). Patients’ clinicopathological characteristics (age, gender, histological type, and pathological TNM staging) are summarized in the “Results” section.

Tissue samples were collected intraoperatively and processed immediately after surgical removal; after the macroscopic visual assessment, the pieces of tumor tissue and unaffected lung tissue from the same lobe or lung of the patient were frozen in liquid nitrogen followed by storage at −80 °C. Prior to processing, the cryo-sections of frozen tissue specimens were stained with hematoxylin–eozyn and evaluated by an experienced pathologists (LC) to confirm the suitability of tumor cell content. Only the tumor samples which contained at least 50 % of tumor cells on a microscopic section as well as unaffected lung tissue samples without malignant cells were used for further processing.

### RNA extraction

Total RNA was isolated from tissue specimens using magnetic extraction method. Briefly, about 40–50 mg of frozen tissue was disrupted in 500 μl of Lysis Buffer (Biomerieux, France) with a TissueRupter (Qiagen, Germany) and incubated with Proteinase K (Sigma-Aldrich, Germany) for 2 h in 56 °C. Nucleic acids from deproteinated cell lysates were extracted automatically on EasyMag machine (Biomerieux, France) according to the producer’s protocol. The 100-μl resulting RNA extracts were stored in −80 °C before further processing.

The concentration of the RNA in the extracts was determined on the NanoDrop 2000c spectrophotometer (Thermo Scientific, UK) and about 500 ng of the RNA was transcripted into cDNA in a reaction with High Capacity RNA-to-cDNA Master Mix (Applied Biosystems, Foster City, CA) according the producer’s recommendations.

### mRNA expression level

An mRNA expression level of 23 genes was evaluated in the tumor and unaffected lung tissues with the comparative real-time PCR (RT-PCR) method using a TaqMan low density array analysis [18]. Ribosomal 18S RNA gene with a relatively low level of the expression variability in lung tissue [9] was used to normalize for the differences in the input cDNA concentration. For each sample, the amplification of all the transcripts was performed simultaneously in the MicroFluid Cards (Applied Biosystems) that contained manufactory loaded and dried commercially available primers/probe sets for gene expression examination (Assays-on-Demand, Applied Biosystems). The Assay-on-Demand accession numbers are summarized in Supplementary Table 1. Each channel of a card was loaded with 100 μl of the reaction mixture containing 50 μl 2XTaqMan Gene Expression Master Mix (Applied Biosystems) and 20 μl of a cDNA solution (corresponding to 100 ng of total RNA). The amplification was performed on a ABI PRISM 7900HT Sequence Detection System equipped with the SDS v.2.4 software for baseline and C_t_ calculations. The cycling conditions were as follows: 50 °C for 2 min followed by 95 °C for 10 min hold and 40 cycles of 95 °C for 15 s and 60 °C for 60 s. Each sample was analyzed in duplicate. To minimize run-to-run variations, the same card was used to evaluate gene expression in tumor and matched normal lung tissues from a particular patient. The raw C_t_ data from each run were exported into the Excel software for further processing. All gene expression measurements were analyzed on the logarithmic scale (log-expression). For each of the 23 analyzed genes, the two measurements were averaged and the ratio, i.e., fold change of the averages for the tumor and normal tissues was computed.

### Statistical analysis

The difference in the log-expression between the tumor and normal tissue (log-fold-change) for each gene was analyzed by using a linear mixed effects model (with a random tissue effect) taking into account the correlation between the repeated expression measurements obtained for a single patient [19]. OS and DFS probabilities were estimated by using the Kaplan-Meier method. The median follow-up time was estimated by using the “reverse” Kaplan-Meier method [20]. The influence of clinical factors and gene expression on OS and DFS was analyzed by using the proportional hazards model. First, a model including age, sex, TNM stage, and histological type was fitted to the data. The model was then simplified by retaining only the factors showing a statistically significant effect. Then, for each gene, the model was extended by including the log-fold-change as a covariate. The effect of the covariate was modeled by using fractional polynomials of order two, which allow flexible modeling of the effect [21]. The statistical significance of the effect was then tested by using the likelihood ratio test.

All the applied tests were two-sided. Given that a number of genes were tested, gene-specific *p* values were corrected for multiple testing by using Hommel’s method [22] with the aim to preserve the overall 5 % level of significance. The analyses were performed by using the SAS 9.2 and STATA 11 statistical software.

## Results

### Patient characteristics

A total of 109 early stage NSCLC patients aged from 39.8 to 78.1 years (mean 61.8, standard deviation 8.4 years) were included in the study. The majority of the patients (77.1 %) were males. Among the patients, 36 (33.0 %) had lung adenocarcinoma (AdC), 48 (44.0 %) had squamous cell carcinoma (SqCC), and the remaining 25 (23.0 %) had large cell carcinoma (LCC) of the lung. Fifty-three (48.6 %) patients had TNM stage I disease: 18 (16.5 %) with stage IA and 35 (32.1 %) with stage IB. Fifty-six patients (51.4 %) had stage II disease: 25 (22.9 %) with stage IIA and 31 (28.5 %) with stage IIB. The median follow-up time was equal to 48.8 months. During the follow-up, 49 patients had disease recurrence and 36 of them had died.

### Differential gene expression between tumor and non-tumor lung tissues

The expression of 23 different genes was analyzed simultaneously in each sample using a TaqMan low density array analysis from Applied Biosystems. We were unable to successfully amplify a number of gene transcripts in some cases possibly due to their tissue level below the detection limit of the assay. As a result, for none of the genes, the log-fold-change could be defined for all the patients. Twenty out of 23 analyzed genes showed statistically significant differences in expression between tumor and non-tumor tissues (Table 2). For 13 genes (*ITGB1*, *ITGB3*, *CXCL1*, *CXCL9*, *CXCL10*, *CXCL12*, *CXCR3*, *CXCR4*, *TNFA*, *CHKA*, *AGFG1*, *SLC2A1*, and *CTC1*), the expression in the tumor was lower than in the normal sample, and for 7 genes (*ITGA5*, *IL8*, *IL6*, *CXCL2*, *CXCL3*, *CXCL5*, and *CXCL11*), an increase in expression in tumor was observed (Table 2).

**Table 2:** Log-fold-changes in gene expression level between tumor and non-tumor lung tissue (the All NSCLC column) and the differences in the log-fold-change between SqCC and LCC (the SqCC vs. LCC column) and between AdC and LCC (the AdC vs. LCC column)

Gene symbol	All NSCLC			SqCC vs. LCC		AdC vs. LCC	
	Samples (*N*)	Log-fold-change^a^ mean ± SD	*p*-value^b^	Log-fold-change difference mean ± SD	*p*-value	Log-fold-change difference mean ± SD	*p*-value
*AGFG1*	98	−0.0298 ± 0.1226	*0.00011*	0.124 ± 0.0278	*0.00004*	0.0564 ± 0.0293	0.11439
*CHKA*	88	−0.0235 ± 0.1371	*0.00004*	0.159 ± 0.0311	*0.00001*	0.0545 ± 0.0322	0.18784
*CXCL1*	70	−0.0438 ± 0.1461	*0.00045*	0.141 ± 0.0324	*0.00006*	0.0695 ± 0.0339	0.08631
*CXCL10*	69	−0.0492 ± 0.1558	*0.00001*	0.179 ± 0.0355	*0.00001*	0.0799 ± 0.0366	0.06389
*CXCL11*	100	0.0040 ± 0.1412	*0.00001*	0.173 ± 0.0386	*0.00004*	0.0700 ± 0.0397	0.16256
*CXCL12*	100	−0.0283 ± 0.1418	*0.00010*	0.163 ± 0.0313	*0.00001*	0.0620 ± 0.0327	0.12277
*CXCL2*	102	0.0613 ± 0.1436	*0.00001*	0.145 ± 0.0322	*0.00004*	0.0343 ± 0.0341	0.63402
*CXCL3*	84	0.0385 ± 0.1357	*0.00001*	0.143 ± 0.0318	*0.00004*	0.0197 ± 0.0334	1.00000
*CXCL5*	74	0.0493 ± 0.1658	*0.01198*	0,122 ± 0.0465	0.02066	0.0355 ± 0.0474	0.91205
*CXCL6*	19	−0.0247 ± 0.1674	0.13503	0.181 ± 0.0719	0.02940	0.1365 ± 0.0763	0.15835
*CXCL9*	93	−0.0723 ± 0.1449	*0.00001*	0.143 ± 0.0342	*0.00013*	0.0546 ± 0.0359	0.26377
*CXCR2*	47	0.0485 ± 0.1626	0.18719	−0.564 ± 0.3973	0.31830	−0.6288 ± 0.4194	0.27431
*CXCR3*	26	−0.0114 ± 0.1613	*0.00122*	0.245 ± 0.0503	*0.00002*	0.1259 ± 0.0510	*0.03375*
*CXCR4*	97	−0.0278 ± 0.1358	*0.00003*	0.154 ± 0.0311	*0.00001*	0.0667 ± 0.0326	0.08614
*IL10*	44	−0.0485 ± 0.1400	0.09305	0.119 ± 0.0448	0.01949	0.0378 ± 0.0460	0.82759
*IL6*	103	0.0403 ± 0.1527	*0.00007*	0.153 ± 0.0349	*0.00005*	0.0585 ± 0.0368	0.22936
*IL8*	101	0.0075 ± 0.1340	*0.02278*	0.111 ± 0.0326	*0.00196*	0.0396 ± 0.0345	0.50733
*ITGA5*	103	0.0085 ± 0.1146	*0.00407*	0.109 ± 0.0265	*0.00015*	0.0448 ± 0.0280	0.22635
*ITGB1*	105	−0.0314 ± 0.1224	*0.00001*	0.140 ± 0.0267	*0.00001*	0.0511 ± 0.0281	0.14398
*ITGB3*	94	−0.0228 ± 0.1660	*0.00005*	0.194 ± 0.0369	*0.00001*	0.0806 ± 0.0388	0.07992
*SLC2A1*	83	−0.1353 ± 0.1320	*0.00001*	0.079 ± 0.0325	0.03216	0.0831 ± 0.0342	0.03384
*CTC1*	88	−0.0189 ± 0.1265	*0.00018*	0.139 ± 0.0296	*0.00002*	0.0565 ± 0.0307	0.13756
*TNFA*	4	−0.0009 ± 0.1956	*0.01730*	0.342 ± 0.0792	*0.00533*	0.1721 ± 0.0548	*0.01745*

Italics indicate *p*-values that are significant while controlling the overall significance level at 0.05

^a^Overall sample mean

^b^ *p*-value based on a model adjusting for histological type

The analysis of the effect of age, gender, TNM stage, or histological type on gene expression revealed that only histological type had substantial influence on the log-fold-change. In particular, for all the genes except of *CXCL5*, *CXCL6*, *CXCR2*, *IL10*, and *SLC2A1*, the effect of histological type was statistically significant at the 5 % significance level (after adjusting the gene-specific *p*-values for multiple testing). In general, for the genes for which histological type had a significant effect, the log-fold-change in SqCC was statistically significantly higher than in AdC or LCC. For the latter two types, the log-fold-change was not statistically significantly different, except of genes *CXCR3* and *TNF*, for which the change was higher for AdC than for LCC. The results are summarized in Table 2.

### Analysis of overall survival

**Fig. 1:**
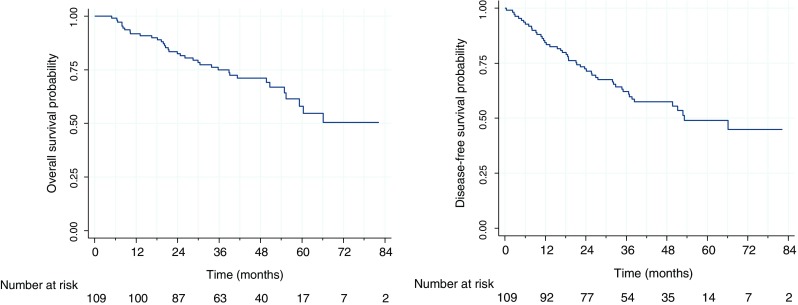
*Left*: The estimated overall survival probability. *Right*: The estimated disease-free survival probability

In the group of 109 analyzed patients, 36 deaths were observed. The median follow-up time was equal to 48.8 months. The left hand side panel of Fig. 1 presents the OS curve for the whole group of analyzed patients.

In the proportional hazards model including patients’ age, gender, TNM stage of the disease (I vs. II), and histological type of tumor, only TNM stage was found to be statistically significantly influencing OS (Table 3). In the model simplified by retaining only TNM, the hazard ratio (HR) for TNM II vs. I was estimated to be equal to 7.5 (95 % confidence interval, CI: [3.09, 18.29]; *p* < 0.001). When the model containing TNM was extended by including the gene-specific log-fold-change for each gene separately as a covariate, only the effect of *CXCL5* was found to be statistically significant (multiple-testing-corrected *p* = 0.04). Five of the genes (*CXCL6*, *CXCR2*, *CXCR3*, *IL10*, and *TNF*) were excluded from the survival analyses due to the lack of expression data for more than 50 % of the patients.

**Table 3:** Multivariable analysis of the prognostic effect of patients’ clinicopathological characteristics on overall survival and disease-free survival (proportional hazards model)

Variable	Overall survival			Disease-free survival		
	Hazard ratio	*p*-value	95 % Confidence interval	Hazard ratio	*p*-value	95 % Confidence interval
Age	1.0057	0.787	0.9652–1.0478	1.0099	0.573	0.9757–1.0453
Gender	0.5145	0.121	0.2221–1.1916	0.6242	0.182	0.3126–1.2466
TNM II	9.8231	*<0.001*	3.7889–25.4677	3.1955	*<0.001*	1.6640–6.1366
AdC	1.3598	0.444	0.6190–2.9874	1.4219	0.297	0.7333–2.7573
LCC	1.9305	0.122	0.8386–4.4440	1.4579	0.321	0.6923–3.0703

Italics indicate *p* values <0.05

**Fig. 2:**
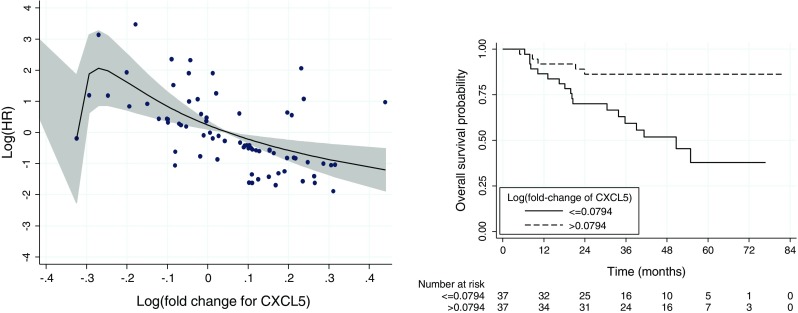
*Left*: The estimated form (*solid line*) of the dependence of the OS log-HR on the log-fold-change of *CXCL5*. The *shaded region* indicates the 95 % CI limits. The *closed circles* indicate deviations from the predicted log-HR curve for individual patients and provide an information about the individual patient values of the log-fold-change. *Right*: The estimated OS curves for two groups of patients with the log-fold-change below or above the observed median (0.0794). The plots are based on data for 74 patients with available information about the expression of the gene

The association between the logarithmically transformed OS hazard ratio and log-fold-change in the *CXCL5* expression between the tumor and normal lung tissue is shown in Fig. 2 (the panel of the left-hand side). The estimated form of the dependence of the log-HR on the log-fold-change of *CXCL5* is presented as a solid line. To provide information about the individual patient values of the log-fold-change, deviations (indicated by the closed circles) from the predicted log-HR curve for individual patients were added to the plot. The sudden drop of the curve at the left hand side is mainly due to a single observation (not shown on the plot) for a TNM IIA patient with the log-fold-change of −0.36, who was alive at the last follow-up visit at around 36 months postsurgery. If we disregard this boundary effect, the plot suggests that the hazard of death continuously decreases with the increasing fold change for the gene. The plot does not suggest any particular threshold value for the log-fold-change.

For illustrative purposes, in the right hand side panel of Fig. 2, we present the OS curves for two groups of patients created arbitrarily by considering the log-fold-change below or above the observed median (0.0794). The curves indicate the higher risk of death for the patients with the log-fold-change below 0.0794, i.e., with the gene expression in the tumor tissue smaller than exp(0.0794) = 1.08 times the expression in the normal tissue.

### Analysis of disease-free survival

In the group of 109 analyzed patients, there were 49 deaths and disease recurrences. Figure 1 (the panel on the right-hand side) presents the DFS curve for the whole group of patients.

In the proportional hazards model including patients’ age, gender, TNM stage (I vs. II) of the disease, and histological type of tumor, only TNM stage was found to statistically significantly influence DFS (Table 3). In the simplified model, HR for TNM II vs. I was estimated to be equal to 2.68 (95 % CI: [1.47, 4.90]; *p* = 0.001).

**Fig. 3:**
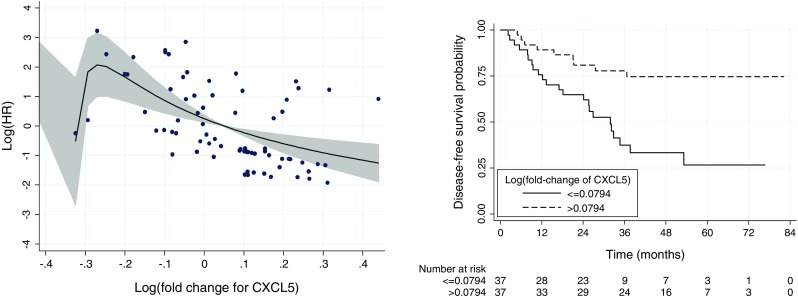
*Left*: The estimated form (*solid line*) of the dependence of the DFS log-HR on the log-fold-change of *CXCL5*. The *shaded region* indicates the 95 % CI limits. The *closed circles* indicate deviations from the predicted log-HR curve for individual patients and provide an information about the individual patient values of the log-fold-change. *Right*: The estimated DFS curves for two groups of patients with the log-fold-change below or above the observed median (0.0794). The plots are based on data for 74 patients with available information about the expression of the gene

Similar to the results of the OS, a statistically significant effect of the log-fold-change, adjusted for the effect of TNM, was found only for gene *CXCL5* (multiple-testing-corrected *p* = 0.004). The left-hand-side panel of Fig. 3 presents the estimated form (solid line) of the dependence of the log-HR on the log-fold-change for *CXCL5*. The shape of the curve is very similar to the one shown in Fig. 3 for OS and suggests that the hazard of disease recurrence continuously decreases with the increasing fold change in the gene expression. For illustrative purposes, in the right hand side panel of Fig. 3, we present the DFS curves for two groups of patients created by considering the log-fold-change below or above the observed median (0.0794). The curves indicate the higher risk of disease recurrence for the patients with the log-fold-change in the *CXCL5* expression below the median value.

## Discussion

The aim of the study was to evaluate the expression of 23 genes as putative prognostic markers in early stage NSCLC. Twenty-three genes had been selected for the examination. These included three genes evaluated by microarray approach (AGFG1/HRB, STC1, and SLC2A1) that overlap between NSCLC prognostic signatures, CHKA suggested as a new prognostic predictor in early stage NSCLC: three genes encoded for intergins α5, β3, and β1 which had been prognostic in the comprehensive analysis of 15 integrin genes by Dingemans et al. [17]: and 16 genes for cytokines or their receptors that have been previously implicated in lung cancer biology [11, 23, 24] and/or demonstrated to be a part of a prognostic signature [12, 15]. A relative quantification RT-PCR method was applied as a research method. The method allows estimation of the fold changes in gene expression between tumor and non-tumor lung tissues from the same patient and is widely used for microarray data verification [4, 25]. To minimize technical, run-to-run variations, a TaqMan low density array analysis that allows a simultaneous examination of the expression of dozens of different genes in each sample was used [5].

In our study, the expression of the majority of the analyzed genes (20 out of 23) in tumor and matched normal lung tissues differed statistically significantly after applying a multiple-testing correction. Among the differentially expressed genes, there were integrin genes *ITGA5*, *ITGB3*, and *ITGB1*; genes for cytokines or their receptors *CXCL1*, *CXCL2*, *CXCL3*, *CXCL5*, *CXCL9*, *CXCL10*, *CXCL11*, *CXCL12*, *CXCR3*, *CXCR4*, *IL6*, *IL8*, and *TNF*; a choline kinase alpha gene *CHKA*; as well as genes *AGFG1* and *CTC1* encoded for nucleoporin-like protein and stanniocalcin-1 (*SLC2A1*), respectively. On the other hand, tumor-associated alterations of the expression level of 3 genes (*CXCL6*, *CXCR2*, and *IL10*) did not reach statistical significance.

Changes in gene expression were more pronounced in SqCC as compared with AdC or LCC. On the other hand, no significant differences in gene expression in tumor related to matched non-tumor lung tissue were found between AdC and LCC samples, except for the activity of *TNF* and *CXCR3*. Based on these results, the analyzed genes seem to be involved in the process of NSCLC evolution, especially in the case of SqCC of the lung, but further examinations are necessary to clarify their contribution to tumor growth and progression.

However, we failed to evaluate the expression of particular genes in some cases possibly due to their tissue level below the detection limit of the assay. Similar results were reported by Seike et al. who had been unable to detect TNF-α and interleukin (IL)-10 expression in a significant percentage of examined tumor and non-tumor tissues [15]. Of interest, the lack of IL-10 expression in specimens from stage I NSCLC patients was found to be associated with significantly worse outcome [16].

One of the major goals of our study was to evaluate the prognostic significance of the tumor-associated alterations in the expression level of the 23 selected genes in stages I and II NSCLCs. In the analyses of OS and DFS, five genes (*CXCL6*, *CXCR2*, *CXCR3*, *IL10*, and *TNF*) had to be excluded from the evaluations due to lack of the expression data for more than 50 % of the patients. For the remaining 18 genes, only the expression of one gene (*CXCL5*) was statistically significantly associated with patients’ overall and disease-free survival after applying a multiple-testing correction (*p* = 0.04 and *p* = 0.004, respectively). Upregulation of *CXCL5* expression in tumor was found to be a favorable prognostic factor for both OS and DFS, and none of the analyzed clinicopathologic characteristics (patients’ age, gender, stage, or tumor histological type) influenced these associations. Gene product CXCL5 (also known as ENA-78/SCYB-5) is a CXC-type chemo-attractive cytokine that was primary described as a powerful attractant for granulocytes and an inflammatory mediator [26]. More recently, CXCL5 contribution to cancer growth and metastasis has been demonstrated [24, 27]. Specifically, CXCL5 has been found to be important in mediating tumor-associated angiogenesis [12, 28] as well as in promoting growth, migration, and invasion of tumorigenic cell lines derived from NSCLC [29] and other cancers [30–32]. The *CXCL5* expression has been shown to be elevated at both mRNA and protein levels and affect patients’ survival in a number of human tumors, including in pancreatic [31, 33], colorectal [34], breast [35], ovarian [36], or prostate [30] carcinomas. However, survival data are limited and inconsistent [31, 37–39]. In the study of White et al., risk of the recurrence after lung cancer resection in NSCLC patients’ was found to be associated with enhanced expression of the sum angiogenic CXC chemokines [12].

In contrast to *CXCL5*, none of the remaining genes examined in our study was found to be associated with patients’ postoperative outcome. It has been repeatedly emphasized that the direct comparison of gene expression data is difficult because potential variations in applied analytical methods and patient selection algorithms may greatly compromise the reproducibility of the results [3, 9, 40, 41]. The analysis of gene expression signatures reported for a defined tumor phenotype or patients’ prognosis reveals very minimal overlapping between them in terms of gene identity. Although a particular signature validation on several independent sample sets generally demonstrated high degree of reproducibility [42], cross-examination of public available gene expression data by different statistical methods generated different results [40, 43]. RT-PCR-based gene expression studies usually used for microarray-based data validation often failed to confirm the examined effect of the gene. Consequently, in the recent critical review of 16 published studies reporting the development of the gene expression-based prognostic signatures in NSCLC, Subramanian and Simon found little evidence that any of the signatures are ready for clinical application [3]. Besides, when effects of multiple genes are investigated, high probability of false-positive finding exists if multiple-testing correction methods are not applied. To illustrate, in the study by Lau et al., who examined 158 putative prognostic genes in a cohort of 147 surgically treated NSCLC patients with RT-PCR method, 24 genes influenced patients’ survival, but the effect of only five of them remained significant after the false discovery rate adjustment for multiple testing [44]. Thus, multiple-testing corrections are very important to properly control the overall probability of a false-positive finding.

In conclusion, in our study, 18 out of the 23 genes previously found to be relevant for NSCLC formation and/or early staged NSCLC patients’ postoperative outcome demonstrated differential expression in tumor and matched unaffected lung tissues. Changes in expression were more pronounced in squamous carcinomas as compared to tumors of non-squamous histology, pointing out the possible contribution of the genes to SqCC carcinogenesis. Among the 23 genes previously suggested to be relevant for early staged NSCLC patients’ postoperative outcome, only *CXCL5* showed a statistically significant prognostic effect.

## References

[1] Strauss GM (2006). Management of early-stage lung cancer: past, present, and future adjuvant trials. Oncology (Williston Park).

[2] Ludwig JA, Weinstein JN (2005). Biomarkers in cancer staging, prognosis and treatment selection. Nat Rev Cancer.

[3] Subramanian J, Simon R (2010). Gene expression-based prognostic signatures in lung cancer: ready for clinical use?. J Natl Cancer Inst.

[4] Provenzano M, Mocellin S (2007). Complementary techniques: validation of gene expression data by quantitative real time PCR. Adv Exp Med Biol.

[5] Goulter AB, Harmer DW, Clark KL (2006). Evaluation of low density array technology for quantitative parallel measurement of multiple genes in human tissue. BMC Genomics.

[6] Beer DG, Kardia SL, Huang CC (2002). Gene-expression profiles predict survival of patients with lung adenocarcinoma. Nat Med.

[7] Raponi M, Zhang Y, Yu J (2006). Gene expression signatures for predicting prognosis of squamous cell and adenocarcinomas of the lung. Cancer Res.

[8] Lu Y, Lemon W, Liu PY (2006). A gene expression signature predicts survival of patients with stage I non-small cell lung cancer. PLoS Med.

[9] Endoh H, Tomida S, Yatabe Y (2004). Prognostic model of pulmonary adenocarcinoma by expression profiling of eight genes as determined by quantitative real-time reverse transcriptase polymerase chain reaction. J Clin Oncol.

[10] Ramirez de Molina A, Sarmentero-Estrada J, Belda-Iniesta C (2007). Expression of choline kinase alpha to predict outcome in patients with early-stage non-small lung cancer: a retrospective study. Lancet Oncol.

[11] McClelland MR, Carskadon SL, Zhao L (2007). Diversity of the angiogenic phenotype in non-small cell lung cancer. Am J Respir Cell Mol Biol.

[12] White ES, Flaherty KR, Carskadon S (2003). Macrophage migration inhibitory factor and CXC chemokine expression in non-small cell lung cancer: role in angiogenesis and prognosis. Clin Cancer Res.

[13] Minamiya Y, Saito H, Takahashi N (2010). Expression of the chemokine receptor CXCR4 correlates with a favorable prognosis in patients with adenocarcinoma of the lung. Lung Cancer.

[14] Suzuki M, Mohamed S, Nakajima K (2008). Aberrant methylation of CXCL12 in non-small cell lung cancer is associated with unfavorable prognosis. Int J Oncol.

[15] Seike M, Yanaihara N, Bowman ED (2007). Use of a cytokine gene expression signature in lung adenocarcinoma and the surrounding tissue as a prognostic classifier. J Natl Cancer Inst.

[16] Soria JC, Moon C, Kemp BL (2003). Lack of interleikin-10 expression could predict poor outcome in patients with stage I non-small cell lung cancer. Clin Cancer Res.

[17] Dingemans A-M, van den Boogaart V, Vosse BA (2010). Integrin expression profiling identifies integrin alpha 5 and beta 1 as prognostic factors in early stage non-small cell lung cancer. Mol Cancer.

[18] Schmittgen TD, Livak KJ (2008). Analyzing real-time PCR data by comparative C_T_ method. Nat Protoc.

[19] Verbeke G, Molenberghs G (2000). Linear mixed models for longitudinal data.

[20] Schemper M, Smith TL (1996). A note on quantifying follow-up in studies of failure time. Control Clin Trials.

[21] Royston P, Sauerbrei W (2008). Multivariable model-building: a pragmatic approach to regression analysis based on fractional polynomials for modelling continuous variables.

[22] Dmitrienko A, Tamhane AC, Bretz F (2010). Multiple testing problems in pharmaceutical statistics.

[23] Vandercappellen J, Van Damme J, Srtuyf S (2008). The role of CXC chemokines and their receptors in cancer. Cancer Lett.

[24] Singh S, Sadanandam A, Singh RK (2007). Chemokines in tumor angiogenesis and metastasis. Cancer Metastasis Rev.

[25] VanGuilder HD, Vrana KE, Freeman WM (2008). Twenty-five years of quantitative PCR for gene expression analysis. BioTechniques.

[26] Walz A, Burgener R, Car B (1991). Structure and neutrophil-activating properties of a novel inflammatory peptide (ENA-78) with homology to interleukin 8. J Exp Med.

[27] Keeley EC, Mehrad B, Strieter RM (2011). Chemokines as mediators of tumor angiogenesis and neovascularization. Exp Cell Res.

[28] Arenberg DA, Keane MP, DiGiovine B (1998). Epithelial-neutrophil activating peptide (ENA-78) is an important angiogenic factor in non-small cell lung cancer. J Clin Invest.

[29] Sun H, Chung WC, Ryu SH (2008). Cyclic AMP-responsive element binding protein- and nuclear factor-kappaB-regulated CXC chemokine gene expression in lung carcinogenesis. Cancer Prev Res (Phila).

[30] Begley LA, Kasina S, Mehra R (2008). CXCL5 promotes prostate cancer progression. Neoplasia.

[31] Li A, King J, Moro A (2011). Overexpression of CXCL5 is associated with poor survival in patients with pancreatic cancer. Am J Pathol.

[32] Kawamura M, Toiyama Y, Tanaka K (2012). CXCL5, a promoter of cell proliferation, migration and invasion, is a novel serum prognostic marker in patients with colorectal cancer. Eur J Cancer.

[33] Frick VO, Rubie C, Wagner M (2008). Enhanced ENA-78 and IL-8 expression in patients with malignant pancreatic diseases. Pancreatology.

[34] Rubie C, Frick VO, Wagner M (2008). ELR + CXC chemokine expression in benign and malignant colorectal conditions. BMC Cancer.

[35] Bièche I, Chavey C, Andrieu C (2007). CXC chemokines located in the 4q21 region are up-regulated in breast cancer. Endocr Relat Cancer.

[36] Furuya M, Suyama T, Usui H (2007). Up-regulation of CXC chemokines and their receptors: implications for proinflammatory microenvironments of ovarian carcinomas and endometriosis. Hum Pathol.

[37] Park JY, Park KH, Bang S (2007). CXCL5 overexpression is associated with late stage gastric cancer. J Cancer Res Clin Oncol.

[38] Okabe H, Beppu T, Ueda M (2012). Identification of CXCL5/ENA-78 as a factor involved in the interaction between cholangiocarcinoma cells and cancer-associated fibroblasts. Int J Cancer.

[39] Speetjens FM, Kuppen PJ, Sandel MH (2008). Disrupted expression of CXCL5 in colorectal cancer is associated with rapid tumor formation in rats and poor prognosis in patients. Clin Cancer Res.

[40] Roepman P, Jassem J, Smit EF (2009). An immune response enriched 72-gene prognostic profile for early-stage non-small-cell lung cancer. Clin Cancer Res.

[41] Skrzypski M (2008). Quantitative reverse transcriptase real-time polymerase chain reaction (qRT-PCR) in translational oncology: lung cancer perspective. Lung Cancer.

[42] Potti A, Mukherjee S, Petersen R (2006). A genomic strategy to refine prognosis in early-stage non-small-cell lung cancer. N Eng J Med.

[43] Singhal S, Miller D, Ramalingam S (2008). Gene expression profiling of non-small cell lung cancer. Lung Cancer.

[44] Lau SK, Boutros PC, Pintilie M (2007). Three-gene prognostic classifier for early-stage non-small-cell lung cancer. J Clin Oncol.

